# Uncovering New Antimelanoma Strategies: Experimental Insights into Semisynthetic Pentacyclic Triterpenoids

**DOI:** 10.3390/life15121884

**Published:** 2025-12-10

**Authors:** Diana Ungureanu (Similie), Larisa Bora, Sorin Dan Chiriac, Ștefana Avram, Daliana Minda, Jevgeņija Lugiņina, Vladislavs Kroškins, Māris Turks, Ioana Zinuca Magyari-Pavel, Ștefania Dinu, Cristina Adriana Dehelean, Corina Danciu

**Affiliations:** 1 Department of Pharmacognosy-Phytotherapy, “Victor Babeș” University of Medicine and Pharmacy Timișoara, Eftimie Murgu Square, No. 2, 300041 Timisoara, Romania; diana.similie@umft.ro (D.U.); stefana.avram@umft.ro (Ș.A.); daliana.minda@umft.ro (D.M.); ioanaz.pavel@umft.ro (I.Z.M.-P.); corina.danciu@umft.ro (C.D.); 2 Research and Processing Center of Medicinal and Aromatic Plants, “Victor Babeș” University of Medicine and Pharmacy Timișoara, Eftimie Murgu Square, No. 2, 300041 Timisoara, Romania; 3 Doctoral School, “Victor Babeș” University of Medicine and Pharmacy Timișoara, Eftimie Murgu Square, No. 2, 300041 Timisoara, Romania; 4 University Clinic of Surgery III, Faculty of Medicine, “Victor Babeș” University of Medicine and Pharmacy Timișoara, Eftimie Murgu Square, No. 2, 300041 Timisoara, Romania; 5 Institute of Chemistry and Chemical Technology, Faculty of Natural Sciences and Technology, Riga Technical University, Paula Valdena Str. 3, LV-1048 Riga, Latvia; jevgenija.luginina@rtu.lv (J.L.); vladislavs.kroskins@rtu.lv (V.K.); maris.turks@rtu.lv (M.T.); 6 Center for Natural and Engineering Sciences, RTU Liepaja Academy, Liela Str. 14, LV-3401 Liepaja, Latvia; 7 Department of Pedodontics, Faculty of Dental Medicine, “Victor Babeș” University of Medicine and Pharmacy Timișoara, No. 9, Revolutiei Bv., 300041 Timisoara, Romania; dinu.stefania@umft.ro; 8 Pediatric Dentistry Research Center, Faculty of Dental Medicine, “Victor Babeș” University of Medicine and Pharmacy Timișoara, No. 9, Revolutiei Bv., 300041 Timisoara, Romania; 9 Department of Toxicology, Drug Industry, Management and Legislation, Faculty of Pharmacy, “Victor Babeș” University of Medicine and Pharmacy Timișoara, Eftimie Murgu Square, No. 2, 300041 Timisoara, Romania; cadehelean@umft.ro; 10 Research Center for Pharmaco-Toxicological Evaluation, “Victor Babeș” University of Medicine and Pharmacy Timișoara, Eftimie Murgu Square, No. 2, 300041 Timisoara, Romania

**Keywords:** betulinic acid, ursolic acid, oleanolic acid, methylphosphonate moieties, melanoma cell line

## Abstract

In view of the concerning rise in the incidence of cancer, natural products are a valuable source for the development of novel therapies. Among phytochemicals, the pentacyclic triterpenoids betulinic (BA), ursolic (UA), and oleanolic (OA) acids, as well as their 3-oxo-derivatives, have attracted considerable attention because of their significant anticancer potential. However, their clinical use is restricted by poor bioavailability, highlighting the need for obtaining semisynthetic derivatives with optimized pharmacokinetic and pharmacodynamic profiles. This study examined the biological effects of twelve new semisynthetic triterpenic and 3-oxo-triterpenic acid derivatives with methylphosphonate moieties of type C(17)-C(O)OCH_2_P(O)(OMe)_2_ and C(17)-C(O)OCH_2_P(O)(ONa)_2_ against the B164A5 murine melanoma cell line. Results have shown that cell viability declined in a dose-dependent fashion, as determined by the MTT assay. In comparison to their parent compounds, derivatives of BoA, OA, OoA, and UoA demonstrated enhanced antiproliferative potential. The Scratch method showed that the anti-migratory effect of all tested compounds was proportional to the dose, while the LDH test indicated no enhanced cytotoxicity relative to the parent compounds. According to the Hoechst 33342 staining, OA derivatives appeared to induce enhanced nuclear condensation signs than the parent compound. Additionally, the HET-CAM assay indicated no evidence of coagulation, hemorrhage, or vascular disintegration. Collectively, these findings suggest that these novel semisynthetic derivatives, particularly OA and OoA derivatives, may be included in future studies on their antimelanoma activity in light of the findings of this preliminary evaluation.

## 1. Introduction

Since ancient times and until present, natural products (NPs) have been used for therapeutic purposes. The use of opium (*Papaver somniferum* L.), myrrh (*Commiphora* spp.), and licorice (*Glycyrrhiza* spp.) has been dated in Mesopotamia since 2600 BC [1]. It is known that around 70,000 plants are used for therapeutic purposes [2]. Penicillin, capsaicin, digitalis glycoside, morphine, codeine, papaverine, and quinine are among the natural molecules currently used in practice in well-established protocols [1,3]. NPs can be used therapeutically in crude form (teas, tinctures, powders), extracts, isolated active compounds (since the 19th century), or as lead molecules to develop semi-synthetic analogs that have improved properties [2]. They represent an extremely valuable source in the discovery of new molecules for the treatment of many diseases due to their extraordinary diversity [4]. Current statistics show that more than 70% of medicines approved by the FDA are derived from natural compounds [5].

Cancer continues to represent a major global health problem, causing more than 16% of deaths worldwide [6]. In 2022, almost 20 million new cases were registered, and approximately 9.7 million patients lost their battle with cancer [6]. It is estimated that by 2050, the number of newly discovered cases will increase to an alarming threshold of 35 million [6]. Melanoma is the most dangerous of skin cancers, and even though it represents less than 5% of the total number, it causes approximately 73% of deaths among skin cancer patients [7,8]. The 5-year survival rate is about 60 to 90% in most regions [9]. Its incidence continues to increase, despite improvements in preventive measures [6]. In Europe, there are 25 cases per 100,000 people [10]. Melanoma originates from melanocytes, the cells from the basal layer of the epidermis that produce melanin, a photoprotective pigment. These cells are primarily found in the skin, but they can also be located in the gastrointestinal/genitourinary tract, eyes, sinuses, and meninges [11,12]. This disease can be presented under 2 clinical subtypes: cutaneous and non-cutaneous, and the cutaneous subtype can also be subclassified into 4 categories: superficial spreading melanoma (70%), nodular melanoma (15–30%), lentigo malignant melanoma (4–10%), and acral lentiginous (<5%) [7]. Acral melanoma has an extremely poor prognosis, leading to metastases in 50% of cases [13]. Risk factors involved in the occurrence of melanoma include: genetic predisposition, phototypes I-II, exposure to UV radiation, immunosuppression, obesity, male sex, and advanced age [7,10,11]. The average age at diagnosis is about 65 years [13]. The genetic changes caused by exposure to intense UV radiation result in DNA alteration and increased oxidative stress, promoting inflammation, which leads to the malignant change in melanocytes [14]. Depending on the stage of the disease, the treatment options are surgical interventions, immunotherapy, radiotherapy, and targeted therapy [11]. Melanoma treatment methods have evolved exponentially in recent decades [14]. Before 2010, only dacarbazine and high-dose interleukin-2 were approved by the FDA for the treatment of metastatic melanoma [14]. After 2010, the FDA approved the use of new therapies, such as immunological agents (ipilimumab, nivolumab, pembrolizumab, and ipilimumab + nivolumab), targeted therapies (vemurafenib, dabrafenib, dabrafenib + trametinib, etc.), but also T-VEC, an intralesional modified oncolytic herpes virus [14]. However, the treatment of melanoma remains a real challenge due to chemoresistance and severe side effects of systemic treatments, so other therapeutic options are still being studied in preclinical and clinical studies [15].

**Figure 1:**
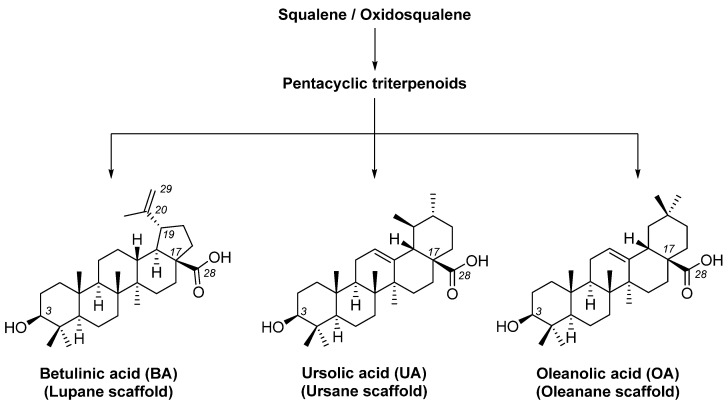
The chemical structures of BA, UA, and OA. The structures were created using ChemDraw 23.0.1.

The diversity of natural products has made them intensively studied in the treatment of cancer in the last half of the current century due to their enhanced safety and effectiveness [16,17]. Among the secondary metabolites of plants, triterpenes, alkaloids, and polyphenolic compounds can exert cytotoxic and chemopreventive activities [18]. Vinblastine, vincristine (*Catharanthus* alkaloids), and paclitaxel are some of the main natural compounds used in the treatment of cancer [4,18]. Between 1981 and 2019, it is estimated that natural products contributed to about 25% of the total number of newly approved anticancer drugs [16]. Triterpenoids represent a vast group of plant metabolites derived from squalene or oxidosqualene (over 20,000 discovered compounds) [19,20,21]. Their chemical structure includes 30 carbon atoms, being composed of isoprene units. Triterpenoids have therapeutic importance due to their proven anti-inflammatory, antioxidant, anticancer, antiviral, antibacterial, and anti-diabetic properties [19,22,23]. They are classified according to the number of rings, the main compounds of therapeutic interest being part of the class of tetracyclic and pentacyclic triterpenoids [24]. Betulinic (BA), ursolic (UA), and oleanolic (OA) acids represent three of the most abundant and studied compounds, belonging to the subclass of pentacyclic triterpenoids (Figure 1). This subclass also includes other compounds of significant interest, such as lupeol, betulin (lupane scaffold), maslinic acid (oleanane scaffold), and uvaol (ursane scaffold) [25].

BA, UA, and OA are potential candidates in the treatment of cancer due to their proven effect against various cancer cell lines (melanoma, leukemia, breast, colon, lung, endometrial, and ovarian cancers) [19,26,27,28,29,30,31]. The vast therapeutic potential of BA, UA, and OA is limited by their poor aqueous solubility, the ability to penetrate cells, respectively, by their low bioavailability, which implies the need to obtain derivatives with superior properties. Numerous semi-synthetic compounds of BA, UA, and OA were obtained by derivatization. BA structure was particularly modified at positions C-2, C-3, C-28, and its C-20/29 olefin. Derivatives with enhanced properties were developed by introducing an amino acid side chain, amine, or hydroxyl groups at C-28, but also by introducing polar sugar moieties at C-3 and/or C-28 [32,33]. Several in vitro studies have demonstrated the effectiveness of BA and its derivatives against certain human and murine melanoma cell lines (A375, SK-MEL28, FM55P, FM55M2, G-361, B164A5) [31,34,35,36]. Furthermore, a BA 20% ointment was tested for treating dysplastic lesions with the potential to progress to melanoma within a phase I/II clinical trial, but the study was stopped due to financial constraints [31].

In the case of UA and OA, the main structural modifications were reported at the C-3, C-28 positions, or both positions simultaneously [5]. Among the structural changes that led to the increase in antiproliferative activity, the transformation of C(17)-COOH into esters or amides or replacing the C(3)-OH group with an oxime moiety can be mentioned [37,38]. Several in vitro studies demonstrated that UA, OA, and the UA + OA combination possess antiproliferative effects against human and murine melanoma cell lines (WM-244-6, MeWo, A375, A375SM, B16) [18,27,39,40]. Also, certain semi-synthetic derivatives of UA and OA have demonstrated their effectiveness against human or murine melanoma cell lines (MeWo, A375, B16-BL6) [27,41]. Moreover, in in vivo studies, OA and its derivative, 3-oxo oleanolic acid, inhibited melanoma tumor growth in murine models (A375SM tumor xenograft model and B16 tumor synografts) [39,41].

As previously mentioned, pentacyclic triterpenoids BA, UA, and OA have potential as valuable anticancer compounds, but this effectiveness is diminished by their extremely poor hydrosolubility [19]. To overcome this issue, existing data suggest that certain modulations can enhance the bioavailability [5]. One potential strategy involves the incorporation of heteroatoms or polar ionogenic groups into the carbon scaffold [42]. Recent research confirms that esterification at C-28 enhances water solubility and bioavailability, respectively, [43]. Another findings suggest that phosphoric and phosphonic acid residues could represent promising functional groups [44]. Moreover, replacing phosphate groups with isosteric and isoelectric phosphonate moieties increases the stability of the compounds [42]. Phosphorus-containing compounds have gained attention in the last 10–15 years due to their potential to activate γδT-lymphocytes, making them in consideration for managing diseases such as cancer, infections, and autoimmune disorders [44]. Many organophosphorus molecules not only have an improved solubility, but also determine an enhanced bio accessibility and transmembrane passage [44]. Regarding the therapeutic molecules that contain phosphonate groups, fosfomycin, alendronate, zolendronate, and tenofovir can be mentioned [44]. The therapeutic potential of triterpene phosphonate derivatives (esters and salts) has not been extensively explored. The investigation of their biological properties is of novelty and practical interest, aiming to obtain derivatives that are more effective than the original compounds.

Insights into the irritation potential of these compounds are important for understanding the mechanisms behind their protective effects against oxidative irritation, as well as for assessing safety and formulation feasibility. The Hen’s Egg Test on the Chorioallantoic Membrane (HET-CAM) assay is a widely accepted in ovo method for assessing irritation potential, particularly for ophthalmic and dermatological formulations, serving as a reliable tool to balance therapeutic efficacy and biocompatibility of active compounds, enhancing the selection of promising compounds for further pharmaceutical development [45].

The objective of this study is to assess the antiproliferative, cytotoxic, anti-migratory, nuclear alteration capacity, and irritation potential of novel methylphosphonate derivatives of BA, UA, and OA (phosphonate methyl esters and phosphonate sodium salts) against the B164A5 murine melanoma cell line.

## 2. Materials and Methods

### 2.1. Compounds and Their Synthesis

Naturally occurring triterpenic acids (betulinic, oleanolic, and ursolic acids) and their 3-oxo-analogs (betulonic, oleanonic, and ursonic acids) were used as standard substances to compare the obtained results. Commercially available oleanolic and ursolic acids were used (purchased from Sigma-Aldrich, St. Louis, MO, USA), but betulinic, betulonic, oleanonic, and ursonic acids were prepared by previously reported oxidative methods [42,46]. Novel triterpenic acid-derived phosphonate methyl esters and phosphonate sodium salts were synthesized and fully characterized in our laboratory by a recently reported procedure [42]. Chemical structures, abbreviations, and common chemical names of all compounds used in this study are provided in Table 1.

### 2.2. Cell Culture

The B164A5 murine melanoma cell line was selected because it is a well-characterized, robust, and reproducible cell model, commonly used for preliminary in vitro assessments [47].

The B164A5 murine melanoma cell line (ECACC 94042254) was provided by Sigma-Aldrich (St. Louis, MO, USA). Under standard conditions (37 °C, 5% CO_2_), the cells were seeded in Dulbecco’s Modified Eagle’s Medium (DMEM) enriched in glucose and fetal calf serum 10% (FCS). To prevent contamination, the medium was enhanced with an association of penicillin and streptomycin (at a concentration of 10,000 IU/mL). Reagents were supplied by Sigma-Aldrich (St. Louis, MO, USA).

### 2.3. Cell Viability Assessment (MTT Assay)

The antiproliferative potential of the natural and semisynthetic compounds was assessed using the MTT (3-(4,5 dimethylthiazol-2-yl)-2,5 diphenyltetrazolium bromide) method, adapted after the protocol of Apostolova et al. [48]. B164A5 cells were seeded into 96-well plates and allowed to stabilize for 24 h, after which they were exposed to BA, BoA, OA, OoA, UA, and UoA derivatives at 1, 10, 25, 50, and 75 μM. Following a 72 h incubation period, each well received 10 μL of MTT solution (5 mg/mL) and the plates were kept for an additional 3h in the incubator to allow formazan crystals formation. The obtained crystals were subsequently solubilized in 100 μL of lysis solution, and the absorbance was measured at 570 nm after 30 min interval using an xMark Microplate Spectrophotometer (BioRad, Hercules, CA, USA). The MTT kit was provided by Sigma-Aldrich (St. Louis, MO, USA).

Half-maximal inhibitory concentrations (IC_50_) values were calculated using GraphPad Prism 8.0.1. The data were analyzed using nonlinear regression with the “Dose-response—Inhibition” model.

### 2.4. Cell Cytotoxicity Assessment (LDH Assay)

The evaluation of the cytotoxic capacity of the derivatives was conducted on murine melanoma cell line (B164A5) cells using the LDH (lactate dehydrogenase) test, based on the procedure Wróblewska-Łuczka et al. outlined [31]. In brief, the cells were cultivated on 96-well plates at a density of 5 × 10^3^ cells/well, and allowed to properly attach for 24 h. They were then exposed to BA, BoA, OA, OoA, UA, and UoA derivatives (25, 50, and 75 μM), then incubated for 72 h. At the end of the treatment period, a volume of 50 μL supernatant from each well was collected and transferred to a separate 96-well plate, and the same amount of reaction mixture was put into each well and left for 30 min at room temperature. Afterwards, 50 μL stop solution was dispensed into all the wells. The level of LDH release was determined at 490, respectively, 680 nm, with measurements performed on an xMark Microplate spectrophotometer (BioRad, xMarkTM Microplate, Serial No. 10578, Tokyo, Japan). The LDH kit was supplied by CyQUANT, Thermo Fisher Scientific (Boston, MA, USA).

### 2.5. Cell Migration Assay (Scratch Method)

The migration potential of B164A5 murine melanoma cells following stimulation with the novel derivatives was assessed utilizing the Scratch method, adapted from Magyari-Pavel et al. [49]. The cells were distributed into 12-well plates at 2 × 10^5^ cells per well and cultured until they reached 90% confluence. A linear scratch was then formed in each well with a sterile tip of a micropipette, after which the cells were gently rinsed with phosphate-buffered saline PBS (Thermo Fischer Scientific, Cambridge, MA, USA) to eliminate detached cellular material and debris. Afterward, the cells were stimulated with BA, BoA, OA, OoA, UA, and UoA derivatives (50 and 75 μM). Images of the wound area were taken immediately after scratching (0 h) and again after one day (24 h) with an Olympus IX73 inverted microscope equipped with a DP74 camera (Olympus, Tokyo, Japan). Cell growth was subsequently assessed using cellSense Dimension software 1.17 (Olympus, Tokyo, Japan). To determine the scratch closure rate, the following formula was implemented:

(1) $$Scratch\;closure\;rate(\%)=\left[\frac{A_{t0}-A_{t}}{A_{t0}}\right]\times 100,$$

where A_t0_ represents the scratch area at the initial time point, and A_t_ represents the area after 24 h.

### 2.6. Hoechst Nuclear Staining

Cell nuclei were highlighted by the Hoechst 33342 staining method as described by Sarău et al. [50]. In the first phase, 10^5^ B164A5 cells/well were stimulated with the natural compounds and their derivatives (50 and 75 μM) for 72 h. Then, the medium was removed. 500 μL of Hoechst solution (1:2000 in PBS) was put into every well, and the cells were placed in a controlled, dark environment, at room temperature for 10–15 min. Afterward, the wells were washed 3 times with PBS. Hoechst 33342 dye was purchased from Thermo Fisher Scientific Inc. (Waltham, MA, USA). Images were captured using a Biotek Lionheart FX automated microscope (Agilent, Santa Clara, CA, USA) and analyzed with Gen5 Microplate Data Collection and Analysis Software(Version 3.14).

### 2.7. HET−CAM Assay

The possible irritation caused by the parent compounds (BA, UA, OA) and their derivatives on chorioallantoic membrane (CAM) tissues was evaluated by applying the HET-CAM protocol [51], with slight adjustments tailored to our experimental setup [52]. This method enabled a semi-quantitative evaluation of the irritation potential of the test compounds on the chorioallantoic membrane of chicken embryos by monitoring vascular reactions right after administration, such as hyperemia, hemorrhage, and coagulation For this purpose, 300 µL of compound solutions, prepared at 75 µM, were administered to the vascularized membrane on the 10th day of embryo development, a stage at which pain perception is absent due to the immature development of neural pathways [53]. Embryonated eggs were randomly assigned to experimental groups using a random number table prior to intervention. The samples were monitored under continuous stereomicroscopic observation for 5 min. Changes in the vascular functionality of the CAM were observed, focusing on abnormal events such as hemorrhage, lysis, and coagulation. After the 5 min observation period, the following formula was used to establish the irritation score:

(2) $$IS=5\times \left[\frac{301-\sec H}{300}\right]+7\times \left[\frac{301-\sec L}{300}\right]+9\times \left[\frac{301-\sec C}{300}\right]$$

where IS denotes the irritation score; secH is the onset time for hemorrhage; secL is for vascular lysis; secC is for coagulation, expressed in seconds.

Vascular alterations of the chorioallantoic membrane (CAM) were analyzed using a Zeiss Discovery 8 stereomicroscope (ZEISS, Göttingen, Germany) fitted with a Zeiss Axio CAM 105 color camera. AxioVision SE64. Rel. 4.9.1 Software (ZEISS, Göttingen, Germany), ImageJ (ImageJ Version 1.50e, https://imagej.nih.gov/ij/index.html, accessed on 21 January 2025), and GIMP software (GIMP v 2.8, https://www.gimp.org/, accessed on 21 January 2025). Pictures were taken both prior to and five minutes following the application of the investigated compounds. Each test group included 6 embryos, following standard practice [54,55]. The results were quantified as irritation score (IS) values calculated using the specified formula. These IS obtained were compared with those of two controls: a negative control (distilled water) and a positive control (0.5% sodium lauryl sulfate, SLS). The interpretation of IS values was based on the Luepke scale [54]: 0–0.9 corresponds to non-irritation, 1–4.9 to slight irritation, 5–8.9 to moderate irritation, and 9–21 to strong irritation. This classification enables the categorization of the tested compounds according to their irritant potential.

### 2.8. Statistical Analysis

GraphPad Prism 8.0.1 was used for data gathering and statistical analysis (GraphPad Software, San Diego, CA, USA). All values are presented as the mean ± standard deviation (SD). To evaluate the statistical differences, one-way ANOVA was conducted, followed by Dunnett’s post hoc test for multiple comparisons. Levels of significance were classified as: * *p* < 0.05; ** *p* < 0.01; *** *p* < 0.001; **** *p* < 0.0001.

## 3. Results

### 3.1. MTT Assay

**Figure 2:**
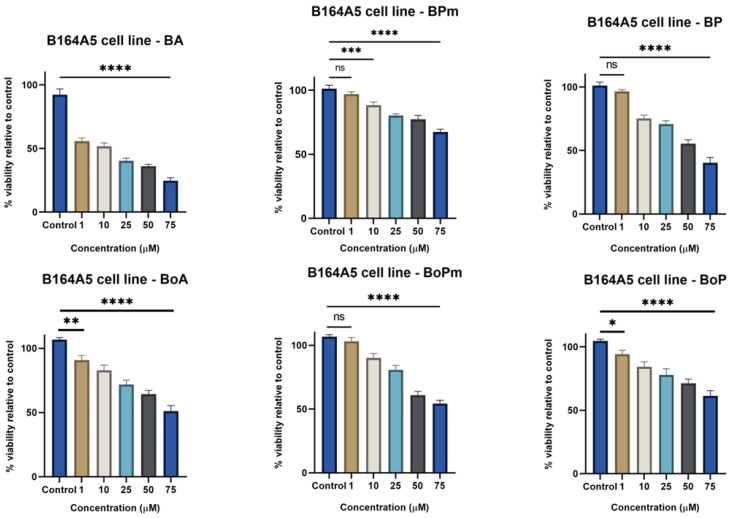
B164A5 murine melanoma cells viability after 72 h stimulation with BA, BPm, BP, BoA, BoPm, and BoP (1, 10, 25, 50, and 75 µM). The results are presented as mean ± SD of three independent experiments. A one-way ANOVA followed by Dunnett’s multiple comparison post-test was performed to compare the groups, with significance levels indicated as follows: * p < 0.05; ** *p* < 0.01; *** *p* < 0.001; **** *p* < 0.0001; ns indicates non-significant differences compared to control cells against control cells.

The cytotoxicity of the compounds was evaluated by the MTT method (3-(4,5-dimethylthiazol-2-yl)-2,5-diphenyltetrazolium bromide) after 72 h of incubation. Betulinic acid (BA), betulonic acid (BoA), and their derivatives were shown to decrease cell viability dose-dependently (Figure 2). IC_50_ values are presented in Table 2. Among BA derivatives, BP displayed a stronger effect than BPm, with IC_50_ values of 57.04 μM. Regarding BoA derivatives, BoPm (IC_50_ = 82.60 μM) showed a more significant antiproliferative effect than BoP (IC_50_ = 108.8 μM) after 72 h of incubation. It was observed that BA derivatives exhibited a weaker antiproliferative effect than BA (IC_50_ = 26.68 μM), indicating that the derivatization process did not enhance the compound’s activity in this case. Similarly, the antiproliferative effect of BoA derivatives was comparable to the parent compound (IC_50_ = 100.60 μM), suggesting that derivatization did not improve the activity in this case. These results suggest that the structural modifications did not markedly enhance the antiproliferative effects, possibly due to limited intracellular activity or a decreased cell penetration of the tested derivatives.

**Figure 3:**
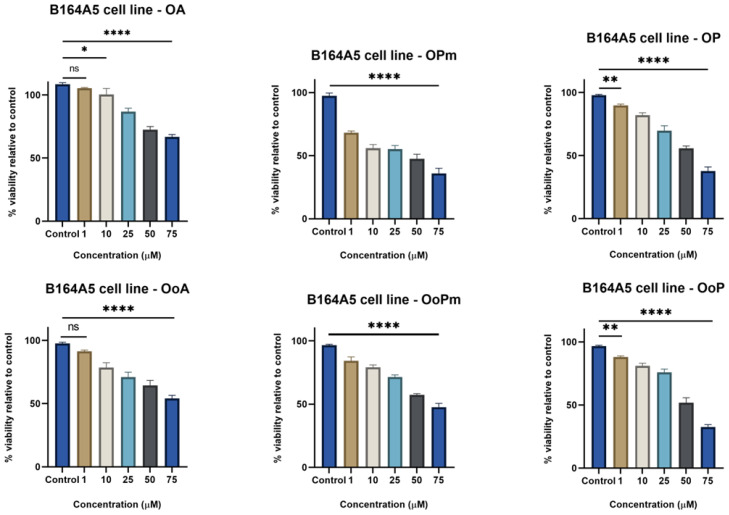
B164A5 murine melanoma cells viability after 72 h stimulation with OA, OPm, OP, OoA, OoPm, and OoP (1, 10, 25, 50, and 75 µM). The results are presented as mean ± SD of three independent experiments. A one-way ANOVA followed by Dunnett’s multiple comparison post-test was performed to compare the groups, with significance levels indicated as follows: * *p* < 0.05; ** *p* < 0.01; **** *p* < 0.0001; ns indicates non-significant differences compared to control cells.

It can also be observed that oleanolic acid (OA), oleanonic acid (OoA), and their derivatives decreased the cell viability in a dose-dependent fashion (Figure 3). Among OA derivatives, OPm displayed a better antiproliferative effect than OP in lower concentrations (10, 25, 50 μM). At 75 μM, both derivatives presented a similar activity, showing satisfactory IC_50_ values of 29.05 µM for OPm and 54.50 µM for OP. Comparing these derivatives with the precursor compound, it can be stated that derivatization improved the antiproliferative effect of OA, especially in the highest concentration (75 μM). Regarding OoA derivatives, OoPm had slightly improved antiproliferative activity than the parent compound; meanwhile, OoP has significantly decreased the B164A5 cell viability at the highest concentration (OoA − IC_50_ = 76.25 μM; OoPm − IC_50_ = 88.92 μM; OoP − IC_50_ = 49.31 μM). Overall, these derivatives displayed an improved antiproliferative effect compared to OA and OoA, proving that the derivatization process was helpful, possibly due to the presence of polar and electron-withdrawing groups that enhanced the interaction between compounds and melanoma cells.

**Figure 4:**
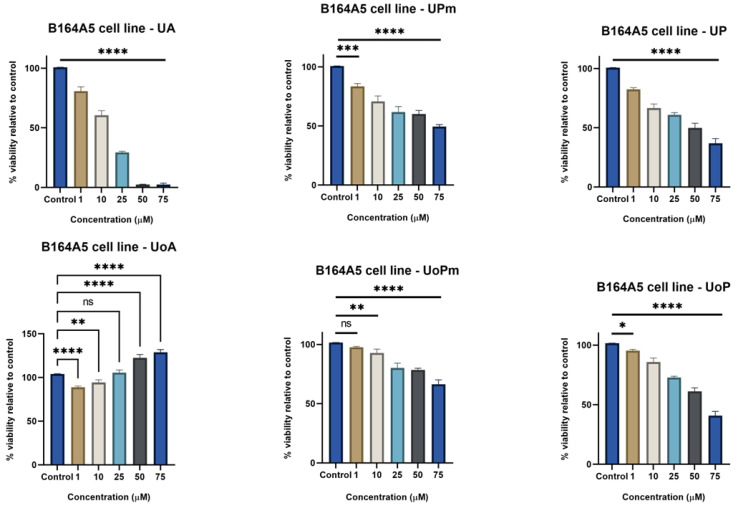
B164A5 murine melanoma cells viability after 72 h stimulation with UA, UPm, UP, UoA, UoPm, and UoP (1, 10, 25, 50, and 75 µM). The results are presented as mean ± SD of three independent experiments. A one-way ANOVA followed by Dunnett’s multiple comparison post-test was performed to compare the groups, with significance levels indicated as follows: * *p* < 0.05; ** *p* < 0.01; *** *p* < 0.001; **** *p* < 0.0001; ns indicates non-significant differences compared to control cells against control cells.

Ursolic acid (UA) and ursonic acid (UoA) had antithetical effects against the B164A5 cell line (Figure 4). While UA induced cell death at 50 and 75 μM (IC_50_ = 12.57 μM), UoA seemed to stimulate cell proliferation. UA derivatives presented a weaker effect than the parent compound. On the other side, UoA derivatives showed an antiproliferative effect, especially UoP at 75 μM (IC_50_ = 62.58 μM). Moreover, between UA and UoA derivatives, UP and UoP determined a more pronounced reduction in cell viability than UPm and UoPm. The contrasting effects of the parent compounds UA and UoA point to a structural influence on the cellular response and bioactivity.

### 3.2. LDH Assay

The lactate dehydrogenase (LDH) assay quantifies the release of lactate dehydrogenase into the medium, which is directly correlated to cell death. Because this method presents limitations in detecting low percentages of LDH, the assay was performed only for the concentrations of 25, 50, and 75 μM, where the decrease in cell viability was more pronounced.

**Figure 5:**
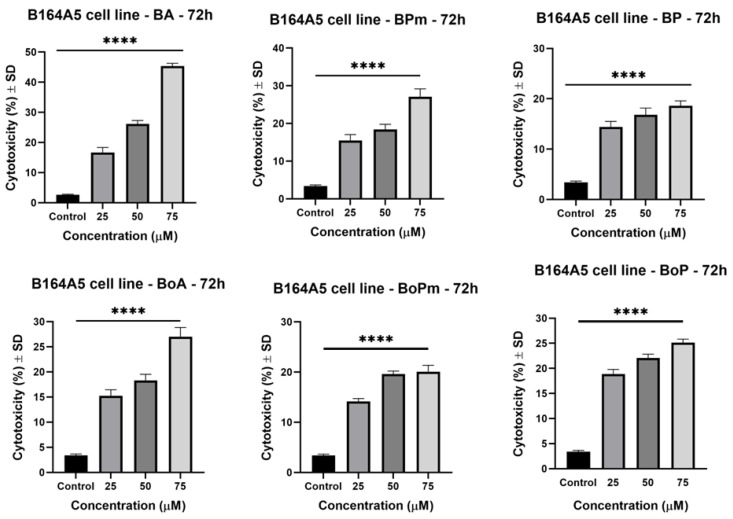
The cytotoxic activity of BA, BPm, BP, BoA, BoPm, and BoP on B164A5 cells (at concentrations of 25, 50, and 75 µM after 72 h of incubation). The results are presented as mean ± SD of three independent experiments. A one-way ANOVA followed by Dunnett’s multiple comparison post-test was performed to compare the groups, with significance levels indicated as follows: **** *p* < 0.0001 vs. control.

It can be observed that BA at 75 μM determined the most substantial LDH release compared to BA derivatives, which suggests that the precursor compound has the strongest cytotoxic effect. At the highest concentration, BoP released an amount of LDH similar to BoA (Figure 5), which suggests that derivatization has not improved the cytotoxic activity.

**Figure 6:**
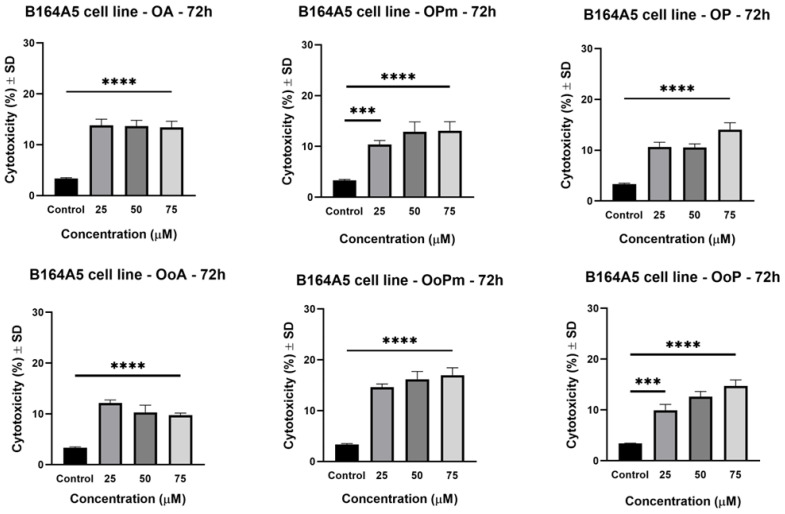
The cytotoxic activity of OA, OPm, OP, OoA, OoPm, and OoP on B164A5 cells (at concentrations of 25, 50, and 75 µM after 72 h of incubation). The results are presented as mean ± SD of three independent experiments. A one-way ANOVA followed by Dunnett’s multiple comparison post-test was performed to compare the groups, with significance levels indicated as follows: *** *p* < 0.001 vs. control, **** *p* < 0.0001 vs. control.

Regarding OA and its derivatives, it can be observed that they presented the weakest cytotoxicity among all of the tested compounds (Figure 6), suggesting a limited ability to cause membrane damage.

**Figure 7:**
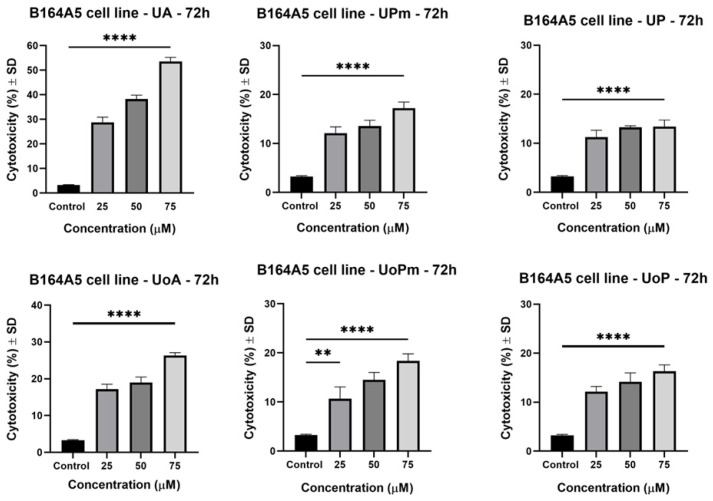
The cytotoxic activity of UA, UPm, UP, UoA, UoPm, and UoP on B164A5 cells (at concentrations of 25, 50, and 75 µM after 72 h of incubation). The results are presented as mean ± SD of three independent experiments. A one-way ANOVA followed by Dunnett’s multiple comparison post-test was performed to compare the groups, with significance levels indicated as follows: ** *p* < 0.01 vs. control, **** *p* < 0.0001 vs. control.

UoA has shown a potent cytotoxic effect at the highest concentration. However, since it seems to stimulate the cell’s viability, the release of LDH could be a consequence of the overpopulation of melanoma cells throughout the 72 h following stimulation. UoA derivatives did not exert significant cytotoxicity (Figure 7).

In agreement with the MTT test, the LDH test demonstrated that UA presented high cytotoxic activity against the B164A5 cell line. Its effect was significantly superior to UA derivatives. In these cases, derivatization did not enhance the cytotoxic activity against the tested cell line.

Although all parent compounds possess a native carboxylic acid group, only BA and UA displayed a strong cytotoxic effect, indicating that the triterpenic scaffold, along with the oxidation state at C-3, may also influence the cytotoxic potential. The results obtained within this method do not represent a limitation, as more active derivatives identified by other techniques may be antimelanoma candidates but act through other mechanisms.

### 3.3. Scratch Assay

**Figure 8:**
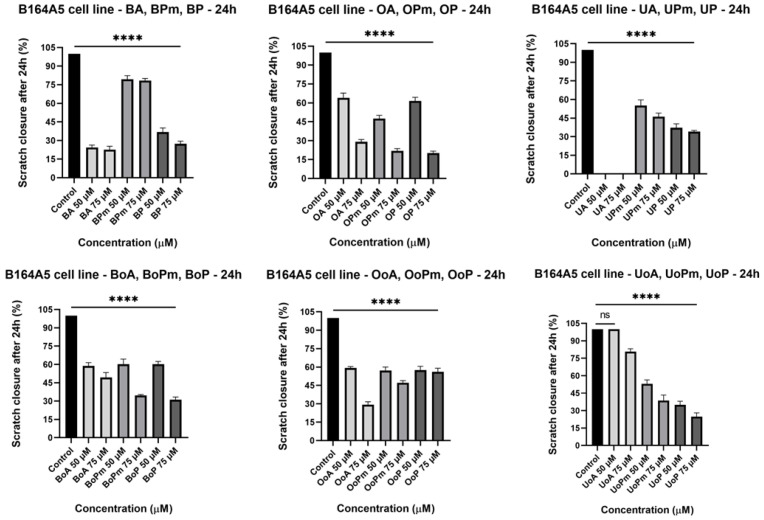
The bar graphs provided a representation of the proportion of wound closure in comparison to the initial surface area measured after 24 h. The results are presented as mean ± SD of three independent experiments. A one-way ANOVA followed by Dunnett’s multiple comparison post-test was performed to compare the groups, with significance levels indicated as follows: **** *p* < 0.0001 vs. control; ns indicates non-significant differences compared to control cells against control cells.

Because of the metastatic behavior of melanoma cells, a wound-healing method was carried out with the aim of evaluating the anti-migratory effect of the novel derivatives, as well as that of the parent compounds. Since better antiproliferative effects were observed at the highest concentrations, the anti-migratory assay was performed for 50 μM and 75 μM doses. The Supplementary Materials section includes microscopy images providing a visual representation of the B164A5 cell migration capacity over 24 h after treatment with the tested compounds. Based on these images, the scratch closure values were further determined. According to Scratch closure after 24 h graphs (Figure 8), all the tested compounds inhibited melanoma cell migration in a concentration-dependent fashion. Thus, regarding BA derivatives, it can be observed that BP exerted a good anti-migratory effect. Further, an improvement in the anti-migratory activity of the BoA derivatives was observed at the highest concentration. In the same manner, OA derivatives present improved anti-migratory effects compared to the parent compound, while OoA derivatives did not show enhanced effects compared to OoA. On the contrary, UA produced an increased cell viability decrease following 24 h of stimulation; therefore, the anti-migratory effect could not be observed due to the extremely potent anticancerous activity against murine melanoma cells. The significant cell loss induced by UA can be directly observed in Figure S5. However, the derivatives exhibited potent migration inhibitory effects, especially at 75 μM. Significant anti-migratory effects were registered for UoA derivatives at both tested concentrations. Concluding, the most potent anti-migratory effects were observed at the dose of 75 μM for the following derivatives: BP, BoPm, BoP, OPm, OP, UP, UoPm, and UoP. Moreover, UP and UoP presented significant wound-healing inhibition rates also at 50 μM. The diverse anti-migratory responses highlight the capacity of some derivatives, such as BoP, UoP, and UoPm, to interfere with melanoma cell movement, possibly through mechanisms distinct from those responsible for their antiproliferative activity, as they did not markedly reduce cell viability.

### 3.4. Hoechst Staining

**Figure 9:**
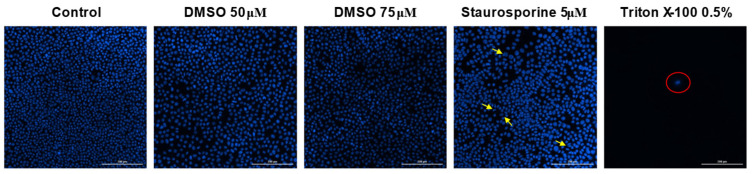
Hoechst 33342 staining of B164A5 cells following the exposure to DMSO (72 h at 50 and 75 µM concentrations). Triton X (0.5%) was utilized to investigate the necrosis process, while Staurosporine (5 μM) served as a positive control to evaluate apoptotic characteristics. The yellow arrows indicate changes related to apoptosis, while the red circle highlights areas of necrosis. A scale bar is equivalent to 200 µm.

**Figure 10:**
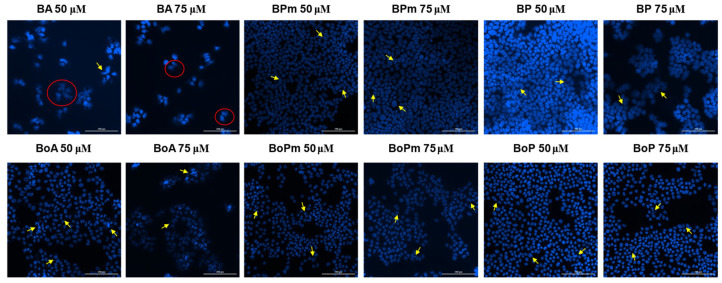
Hoechst 33342 staining of B164A5 cells following the exposure to BA and BoA, respectively, of their derivatives (72 h at 50 and 75 µM concentrations). The yellow arrows indicate changes related to apoptosis, while the red circle highlights areas of necrosis. A scale bar is equivalent to 200 µm.

**Figure 11:**
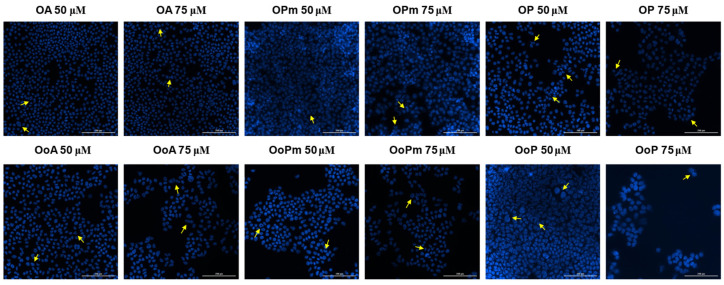
Hoechst 33342 staining of B164A5 cells following the exposure to OA and OoA, respectively, of their derivatives (72 h at 50 and 75 µM concentrations). The yellow arrows indicate changes related to apoptosis. A scale bar is equivalent to 200 µm.

**Figure 12:**
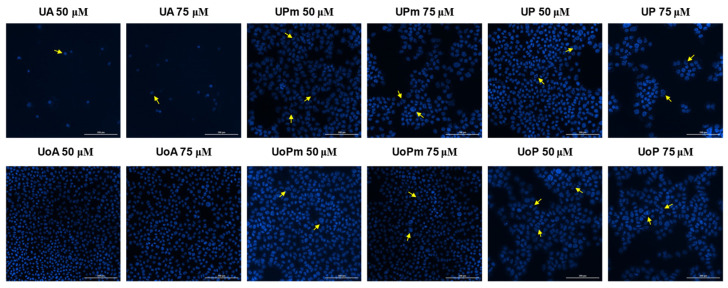
Hoechst 33342 staining of B164A5 cells following the exposure to UA and UoA, respectively, of their derivatives (72 h at 50 and 75 µM concentrations). The yellow arrows indicate changes related to apoptosis. A scale bar is equivalent to 200 µm.

Hoechst 33342 staining was further employed to visualize nuclear morphology changes and to obtain preliminary insights regarding the mechanism of action of the tested compounds. Therefore, cell nuclei morphology was evaluated following stimulation with the eighteen tested compounds, and also for the solvent (DMSO), to exclude any potential cytotoxic effect. Similarly, only the highest concentrations were tested (50 μM and 75 μM). DMSO exerted no cytotoxic effect on the melanoma cell line (Figure 9). BA induced nuclear features suggestive of necrosis at both tested concentrations (Figure 10). BPm induced slight signs of apoptosis features, while BP seemed to affect the nuclei morphology at 75 μM, where chromatin condensation can be observed. For BoA (75 μM) and its derivatives, chromatin condensation indicative of apoptosis features was observed. Regarding OA and OoA, the staining revealed that the derivatives showed more pronounced chromatin condensation than the parent substances, suggesting a stronger potential to induce apoptosis features (Figure 11). UA turned out to be the most potent compound and induced nuclear features consistent with necrosis of B164A5 cells, while the derivatives caused features consistent with apoptosis (Figure 12). On the opposite, signs of apoptosis or necrosis were not present following UoA treatment. Poor apoptosis characteristics appeared in the case of the UoPm derivative, while the UoP derivative appeared to induce specific features of apoptosis.

However, since Hoechst staining alone cannot reliably distinguish apoptosis from necrosis, further mechanistic studies are necessary to confirm the type of cell death.

### 3.5. Irritative Potential in Ovo, Using the HET-CAM Assay

**Figure 13:**
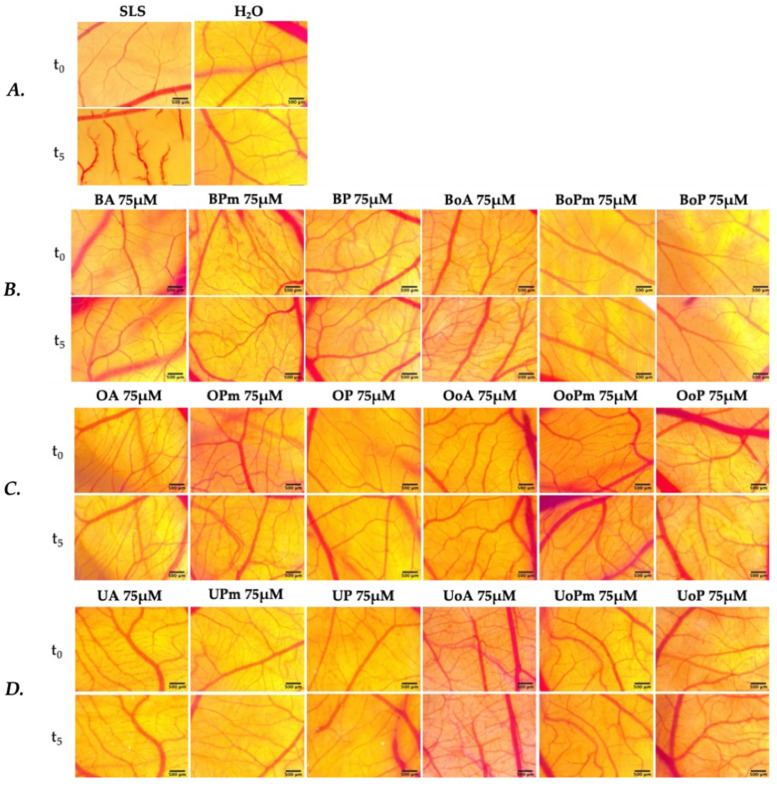
Effects of the tested UA, OA, BA, and their derivatives in the HET-CAM assay: (**A**) Representative CAM images for SLS (positive control) and H_2_O (negative control); (**B**) Representative CAM images for BA, BoA, and their derivatives; (**C**) Representative CAM images for OA, OoA, and their derivatives; (**D**) Representative CAM images for UA, UoA, and their derivatives. Stereomicroscope images show the aspect of CAMs before (t_0_), and after 300 s (t_5_); scale bars represent 500 µm.

The application of samples containing BA, BPm, BP, BoA, BoPm, BoP, OA, Opm, OP, OoA, OoPm, OoP, UA, UPm, UP, UoA, UoPm, UoP did not result in any changes to the vascular parameters assessed. In contrast to the positive control, SLS, which caused significant irritation (IS = 15.74 ± 0.52), the tested samples were well tolerated and showed no irritative effects on the chorioallantoic membrane, similar to the negative control, distilled water (Figure 13, Table 3). No sign of coagulation, hemorrhage, or vascular disintegration was registered during the evaluation of all derivatives at the highest tested concentration of 75 μM. The irritation score (IS) for each sample was calculated using the standard HET-CAM protocol equation [54]. Since no vascular reactions were detected within the 300 s observation window, all onset times were >300 s, yielding IS = 0 for every derivative. This confirms the complete absence of acute vascular irritation, a finding further supported by microscopic evaluation (Figure 13), which revealed intact vasculature in all treated CAMs.

Samples were classified according to the standard HET-CAM irritation scale (non-irritant, slight, moderate, strong irritant). All triterpenoid derivatives were categorized as non-irritant, while the positive control (0.5% SLS) was classified as a strong irritant, thus confirming both the favorable biocompatibility of the tested triterpenoids and the sensitivity of the assay.

## 4. Discussion

The viability of B164A5 cells was assessed after exposure to the parent compounds (BA, BoA, OA, OoA, UA, UoA) and their derivatives. Cell viability declined in a dose-dependent manner following exposure to the investigated compounds 72 h after stimulation, the best effects being observed at the highest concentrations (50 μM and 75 μM). Further, since better antiproliferative effects were observed at the highest concentrations, the cytotoxicity assay was conducted for 25 μM, 50 μM, and 75 μM, while the anti-migratory and pro-apoptotic effects were tested for 50 μM and 75 μM doses. All tested assays demonstrated a dose-dependent effect of the compounds, with some of them exhibiting greater activity than their parent molecules. Furthermore, this study included a first preliminary evaluation of the pro-apoptotic effects of the semisynthetic derivatives with the help of the Hoechst 33342 staining. The findings indicate that the pro-apoptotic potential deserves further investigation; the objective of the next study will be to evaluate the molecular mechanism of action of the active compounds. Some preliminary results acquired from initial experiments regarding BA derivatives were previously briefly described by Similie et al. [56].

The scientific literature confirms the significant anticancer potential of betulinic acid (BA) and betulonic acid (BoA) against various cancer cell lines, including melanoma [57]. In this study, the derivatization process did not improve the anticancerous potential of the four new derivatives against murine melanoma cells. However, a slightly better antiproliferative effect was observed for the BoPm derivative compared to its parent compound (IC_50_ values of 82.6 μM, respectively, 100.6 μM). Many research studies demonstrated the antiproliferative effects of BA on different melanoma cell lines. In this regard, Soica et al. showed that 10 mM BA diminished B164A5 cell proliferation after an exposure of 72 h by approximately 50% [58]. Farcas et al. observed the same trend: 25 μM BA decreased B164A5 cell viability by 40% after 48 h of exposure [59]. These results confirm the efficient and potent effect of BA and BoA against melanoma cells. Therefore, considerable research efforts are directed toward identifying terpene derivatives with improved pharmacodynamic, pharmacokinetic, and pharmacotoxicological characteristics. Lombrea et al. studied the effects of a series of BA semisynthetic derivatives (indole-conjugates) against two melanoma cell lines (B164A5 and A375) and obtained promising results. *N*-(2,3-indolo-betulinoyl)glycylglycine and *N*-(2,3-indolo-betulinoyl)glycine compounds exhibited the most satisfactory results in terms of antiproliferative, cytotoxic, anti-migratory, and pro-apoptotic activities against the B164A5 cell line [60]. Meanwhile, *N*-(2,3-indolo-betulinoyl)diglycylglycine demonstrated superior melanoma activity compared to *N*-(2,3-indolo-betulinoyl)glycine against A375 cells [61]. Drąg-Zalesińska et al. evaluated five amino acid ester derivatives of betulin against the ME-45 human metastatic melanoma cell line and concluded that the derivatives showed better antimelanoma effect than their precursors, due to the improved pharmacokinetic properties (better solubility, thus better bioavailability) and increased concentration in cancerous cells [62]. Yang et al. obtained nineteen betulin derivatives esters and tested them against several cancer cell lines, including A375 human melanoma cells. Promising results were observed for some of the derivatives, from which succinyl-extended piperidine C(17)C(O)O-(CH_2_)_2_-C(O)N(CH_2_)_5_ was the most potent on all the tested cell lines. MTT assay revealed IC_50_ values ranging from 4.3 to 7.5 μM after 72 h of exposure (IC_50_ of 7.5 μM for A375 cells). Moreover, it exhibited selective cytotoxic activity on the cancerous cells, being less toxic to the normal ones (NIH3T3 mouse embryo fibroblast cell line) [63]. Although several studies highlight enhanced anticancer activity for various BA derivatives, our findings indicate that the derivatives did not surpass the parent compounds in terms of antiproliferative and cytotoxic effects against the B164A5 cells. This outcome reflects the strong dependence of biological activity on the structural modifications introduced in the lupane scaffold.

As depicted in Section 3, OA and OoA derivatives demonstrated superior antiproliferative effects compared to the original compounds, indicating the effectiveness of the derivatization process in this case. These facts are also supported by the IC_50_ values (Table 2). Despite their improved antiproliferative activity, these derivatives did not cause substantial LDH release, suggesting limited membrane damage. This may reflect a possible cytostatic rather than a cytotoxic effect, indicating that the compounds may interfere with the cellular metabolism or proliferation instead of inducing direct cell membrane lysis. Therefore, a necrotic cytotoxic response appears to be excluded. Similar differences between antiproliferative assays (e.g., MTT, Neutral Red) and LDH results have been reported in the literature, where the samples affected cell proliferation without causing direct cell membrane damage [64,65]. Woo et al. reported that OA (20, 40, 60, 80, and 100 μM) decreased the viability of A375P and A375SM human melanoma cells in a dose-dependent manner and induced apoptosis, as shown by DAPI staining and flow cytometry. The researchers concluded that OA triggers apoptosis via the NF-κB pathway [39]. On the contrary, Oprean et al. demonstrated that OA did not induce a significant apoptotic effect on B164A5, a slight effect being observed at the highest tested doses (75 μM and 100 μM) [66]. The results of Oprean et al. are consistent with those observed in the current study. It seems that OA-induced apoptosis is cell-line dependent, with murine melanoma cells being less affected compared to human melanoma models. Further, Bednarczyk-Cwynar et al. evaluated the activity of OA and their derivatives (oleanonic acid-derived C(28)-morpholinamides bearing *O*-acylated-3-oximes) on two human melanoma cell lines (MeWo and A375). MTT and SRB assays provided data regarding the antiproliferative effect of the tested compounds, with the observation that all the substances displayed an effect in a dose-dependent manner. After 48 h of treatment, the derivatives significantly reduced MeWo cell viability at the highest tested concentration (100 μM), with *O*-(2-bromoacetyl) 3-oxime derivative being the most potent (10.9% versus other derivatives in the range 37.2–81.2%), while OA did not reduce cell viability. The results obtained using the SRB assay displayed a similar trend. On the other hand, different results were obtained on A375 cells. OA and acyloxime derivative did not produce any toxic effect on this human melanoma cell line following the SRB assay. However, the MTT assay in the paper by Bednarczyk-Cwynar et al. showed that the 48 h-treatment at 100 μM did influence A375 viability (16–48.65% for 3-oxime-28-amide derivatives (50 μM); and 61% for OA). The authors affirmed that the anticancerous effect was improved in OA derivatives, especially in the alkyl derivatives, with *O*-acetyl-3-oxime and *O*-(2-bromoacetyl) 3-oxime derivatives being the most active on both tested human melanoma cell lines [27]. Another study highlights the potent antimelanoma effect of a 3-oxo-OA derivative. The anticancerous impact of the derivative was tested on numerous cancer cell lines, including murine melanoma cell lines B16-BL6 and B16 [41].

Regarding UA and UoA derivatives, the results lead to the conclusion that the derivatization process was ineffective in improving the anticancer effect of UA derivatives. UA induced marked cytotoxicity and nuclear changes consistent with necrosis, while the derivatives produced milder effects, potentially associated with apoptosis. This difference may suggest that the structural modifications produced a more controlled response of cell death, namely the apoptotic pathway. However, UoA derivatives exerted a better anticancer effect than the parent compound (UoA seemed to stimulate the proliferation of B164A5 cells), but still not a remarkable one for the UoPm derivative, while UoP exerted a potent effect at 75 μM. UoA exhibited a mild metabolic stimulatory response in B164A5 cells, leading to viability values higher than those of the control cells. As the MTT assay measures cell viability by quantifying mitochondrial metabolic activity, compounds with stress-adaptive properties may mildly enhance mitochondrial function, consequently increasing the MTT signal. Therefore, the observed effect may reflect a stress-adaptive response induced by UoA [67]. These findings align with the existing literature, as specific derivatives can either enhance or reduce the cytotoxic profile based on the modulations made. Oprean et al. tested the anticancerous effect of UA on both a murine and a human melanoma cell line (B164A5 and A375). As expected, UA exerted a dose-dependent antiproliferative effect on both cancer cell lines, with slightly better activity on the murine cells. Moreover, the compound caused changes in the cell cycle, leading to cell arrest in the G0/G1 phase. The researchers also employed Annexin V apoptosis assay and established that UA induces significant apoptosis in both tested melanoma cell lines, with a decrease in *bcl-2* gene expression [66]. Thirteen UA derivatives were synthesized and further analyzed by Yang et al. The most potent derivative was compound 5b (*N*-[3b-acetoxy-urs-12-en-28-oyl]-amino-piperazine). When tested against melanoma cell lines, it determined strong antiproliferative effects (MTT assay), with IC_50_ values of 4.79 ± 0.73 μM for the B16-F10 cell line, respectively, 6.26 ± 0.38 μM for the A375 cell line. The obtained results emphasize that the incorporation of the piperazine moiety at C-28 was successful [68]. In the same vein, Kahnt et al. tested a series of UA derivatives against several cancer cell lines, including the A375 human melanoma cell line. Following the SRB assay after 72 h of treatment, piperazine-spacered 1,4,7,10-tetraazacyclododecane-1,4,7,10-tetraacetic acid (DOTA) conjugate revealed the strongest cytotoxic activity against A375 melanoma cells (EC_50_ = 1.5 ± 0.4 μM). Further, the fluorescence microscopy evaluation and Annexin V-FITC/PI assay showed ruptures of the cell membrane and signs of necrotic and apoptotic cells after treatment for 48 h with this compound. Also, a reduction in G1/G0, G2/M, and S phases was observed following cell cycle evaluation [69]. 3-*O*-acetyl-ursolic acid derivative was tested by AlQathama et al. in terms of antiproliferative, anti-migratory, anti-invasive, and pro-apoptotic activities on both human and murine melanoma cell lines. SRB assay showed that both the derivative and parent compound presented dose-dependent antiproliferative effects against the A375 cell line, with IC_50_ values of 32.4 ± 1.33 μM and 26.7 ± 3.61 μM, respectively. Moreover, both tested compounds triggered signs of early apoptosis, increased caspase-3 activity, and shifted the balance between Bax and Bcl-2 gene expression in favor of Bax, thus engaging mitochondrial-related apoptosis. Interestingly, 3-*O*-acetyl-ursolic acid showed no anti-migratory capacity against B16F10 murine melanoma cells, while UA did exhibit this effect. This suggests that the hydroxyl group may be essential for the anti-migratory effects [70]. Scientific literature indicates that significantly fewer studies have investigated the anticancer effects of ursonic acid, especially on melanoma cell lines. According to Son et al.’s review, UoA presents an anticancer effect on SK-MEL-2, SK-MEL-28, and B16F10 malignant melanoma cell lines [71].

Although all the tested compounds were derivatized in a similar manner, the differences in the biological effects suggest that the triterpenic scaffold plays an important role in modulating the effects against the B164A5 cell line.

The HET-CAM assay was used to evaluate the irritation potential of BA, UA, OA, and their derivatives against a biological model (the chick embryo chorioallantoic membrane—CAM). The HET-CAM assay serves as a reliable and ethical alternative to traditional Draize rabbit eye and skin irritation tests, aligning with the 3Rs principles (Interagency Coordinating Committee on the Validation of Alternative Methods (ICCVAM), ICCVAM-Recommended Test Method Protocol: Hen’s Egg Test-Chorioallantoic Membrane (HET-CAM) Test Method. NIH Publ. No. 10-7553–Publ. 2010. Available online: https://ntp.niehs.nih.gov/sites/default/files/iccvam/docs/ocutox_docs/invitro-2010/tmer-vol1.pdf (accessed on 12 February 2025). The assay involved the contact between the test compounds and CAM by observing the sensitivity to inflammatory responses induced in the conjunctival blood vessels, such as hemorrhage, lysis, and coagulation, making it valuable for early irritation screening. Based on the Luepke score, the irritant potential of each compound was determined. As can be seen, none of the tested compounds at the concentration of 75 μM presented any irritating potential (IS = 0), which is consistent with other data available in the literature. Regarding the BA derivatives, Milan et al. synthesized fatty acid esters of betulinic acid using butyric, stearic, and palmitic acids and then evaluated their irritative capacity. These derivatives were found to be non-irritating (IS = 0) [72]. As for other triterpene derivatives, Kazakova et al. synthesized some novel 3-pyridinylidene derivatives of chemically modified lupane and ursane triterpenes. The most potent anticancer derivatives (3-oxo-2-(3-pyridinylidene)-21-[3-(2*E*-pyridinyl)-prop-2-en-1-one]-20β,28-epoxy-18aα,19βH-ursane and methyl 3,20-dioxo-2-(3-pyridinylidene)-29-nor-lup-28-oate) were also evaluated for their irritant potential. The results of the HET-CAM test indicated an IS of 0 for both derivatives, placing them in the category of non-irritating compounds [73]. Within another similar study, some novel synthesized N-ethyl-piperazinyl-amides of C2-substituted OA and UA were investigated. These derivatives also displayed no irritant potential, with an IS of 0 [74].

Excessive irritation could limit formulation options such as topical delivery for melanoma treatment. The lack of irritative effect of the tested triterpenic compounds and their derivatives is therefore a valuable trait for future topical delivery systems.

As this was a preliminary investigation involving a large number of active compounds, this study was limited to a single melanoma cell line. Future research will involve the biological evaluation of the most active derivatives on additional melanoma cell lines (both human and murine) and will aim to elucidate the underlying molecular mechanisms of action.

## 5. Conclusions

Twelve novel methylphosphonate derivatives of betulinic, oleanolic, ursolic acids, and their 3-oxo-congeners were biologically evaluated in the murine melanoma cell line B164A5 model. The in vitro tests revealed that all of them displayed a dose-dependent effect. BA derivatives were found to be inferior in terms of biological activity to the parent compound, even if BP displayed good anti-migratory activity. Regarding the UA derivatives, the phosphonate salts (UP and UoP) displayed a better antiproliferative and anti-migratory potential than the phosphonate methyl esters (UPm and UoPm), but their overall anticancer effect was inferior to the natural compound. Nevertheless, the derivatization process of oleanolic acid was found to be rewarding, leading to compounds with superior antimelanoma properties, especially for compounds OP, OPm, and OoP. Importantly, none of the new derivatives exhibited any irritating effects on the chick embryo chorioallantoic membrane. This preliminary study helped identify the most active derivatives on the screened melanoma cell line. Further in vitro and in vivo research is necessary to validate the anticancer potential of these semisynthetic derivatives and to elucidate the underlying molecular mechanism of action.

## Figures and Tables

**Table 1:** Natural and semi-synthetic pentacyclic triterpenoids used in this study.

Natural Product Class	Natural Product Standard	Semi-Synthetic Phosphonate Methyl Ester	Semi-Synthetic Phosphonate Sodium Salt
Lupane type triterpenoids	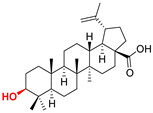 **BA** Betulinic acid CAS: 472-15-1	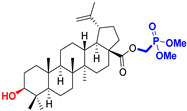 **BPm** (17 S)-17-(((dimethoxyphosphoryl)methoxy)carbonyl)-3β-hydroxy-28-norlup-20(29)-ene	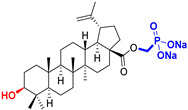 **BP** sodium (3β-hydroxy-(17R)-17-28-norlup-20(29)-en)-2-oxoethyl-phosphonate
	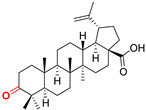 **BoA** Betulonic acid CAS: 4481-62-3	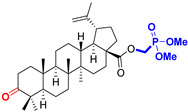 **BoPm** 3-oxo-(17 S)-17-(((dimethoxyphosphoryl)methoxy)carbonyl)-28-norlup-20(29)-ene	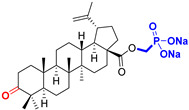 **BoP** sodium (3-oxo-(17R)-17-28-norlup-20(29)-en)-2-oxoethyl-phosphonate
Oleanane type triterpenoids	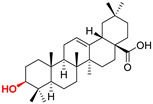 **OA** Oleanolic acid CAS: 508-02-1	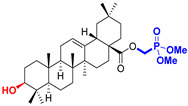 **OPm** (17 S)-17-(((dimethoxyphosphoryl)methoxy)carbonyl)-3β-hydroxy-28-norolean-12(13)-ene	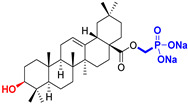 **OP** sodium (3β-hydroxy-(17R)-17-28-norolean-12(13)-en)-2-oxoethyl-phosphonate
	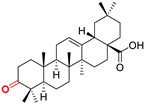 **OoA** Oleanonic acid CAS: 17990-42-0	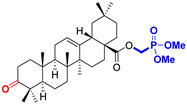 **OoPm** 3-oxo-(17 S)-17-(((dimethoxyphosphoryl)methoxy)carbonyl)-28-norolean-12(13)-ene	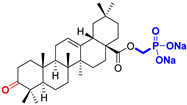 **OoP** sodium (3-oxo-(17R)-17-28-norolean-12(13)-en)-2-oxoethyl-phosphonate
Ursane type triterpenoids	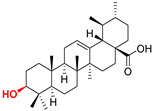 **UA** Ursolic acid CAS: 77-52-1	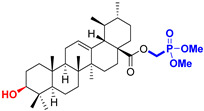 **UPm** (17 S)-17-(((dimethoxyphosphoryl)methoxy)carbonyl)-3β-hydroxy-28-norurs-12(13)-ene	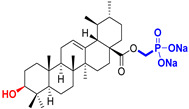 **UP** sodium (3β-hydroxy-(17R)-17-28-norurs-12(13)-en)-2-oxoethyl-phosphonate
	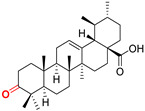 **UoA** Ursonic acid CAS: 6246-46-4	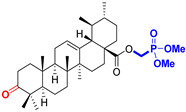 **UoPm** 3-oxo-(17 S)-17-(((dimethoxyphosphoryl)methoxy)carbonyl)-28-norurs-12(13)-ene	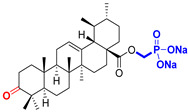 **UoP** sodium (3-oxo-(17R)-17-28-norurs-12(13)-en)-2-oxoethyl-phosphonate

**Table 2:** IC_50_ values (μM) of triterpenic acids and their methylphosphonate derivatives against B164A5 murine melanoma cells. Bolded values represent the derivatives with improved antiproliferative activity compared to their corresponding parent compounds.

Triterpenic Acid C(17)-COOH Series (μM)		Triterpenic Methylphosphonate C(17)-C(O)OCH_2_P(O)(OMe)_2_ Series (μM)		Triterpenic Methylphosphonate C(17)-C(O)OCH_2_P(O)(ONa)_2_ Series (μM)	
BA	26.68	BPm	143.70	BP	57.04
BoA	100.60	**BoPm**	**82.60**	BoP	108.8
OA	118.9	**OPm**	**29.05**	**OP**	**54.50**
OoA	76.25	OoPm	88.92	**OoP**	**49.31**
UA	12.57	UPm	55.35	UP	40.94
UoA	-	UoPm	199.9	**UoP**	**62.58**

**Table 3:** Irritation scores and the types of effects investigated through the HET-CAM test for the evaluation of UA, OA, BA, and their derivatives.

Samples	Irritation Score (IS)	Type of Effect
SLS 0.5%	15.74 ± 0.52	strong irritant
H_2_O dist.	0 ± 0	non-irritant
All tested pentacyclic triterpenoids (BA, BPm, BP BoA, BoPm, BoP OA, OPm, OP OoA, OoPm, OoP UA, UPm, UP UoA, UoPm, UoP)	0 ± 0	non-irritant

## Data Availability

Data are contained within the article and Supplementary Materials. Further inquiries can be directed to the corresponding authors.

## Supplementary Materials

The following supporting information can be downloaded at: https://www.mdpi.com/article/10.3390/life15121884/s1, Figure S1: Anti-migratory activity of BA, BPm, and BP; Figure S2: Anti-migratory activity of BoA, BoPm, and BoP; Figure S3: Anti-migratory activity of OA, OPm, and OP; Figure S4: Anti-migratory activity of OoA, OoPm, and OoP; Figure S5: Anti-migratory activity of UA, UPm, and UP; Figure S6: Anti-migratory activity of control, UoA, UoPm, and UoP.
