# Supplementary figures and images for: Uncovering New Antimelanoma Strategies: Experimental Insights into Semisynthetic Pentacyclic Triterpenoids
Source: Life (Basel). 2025 Dec 10;15(12):1884. doi: 10.3390/life15121884 (PMC12734932; doi:10.3390/life15121884)

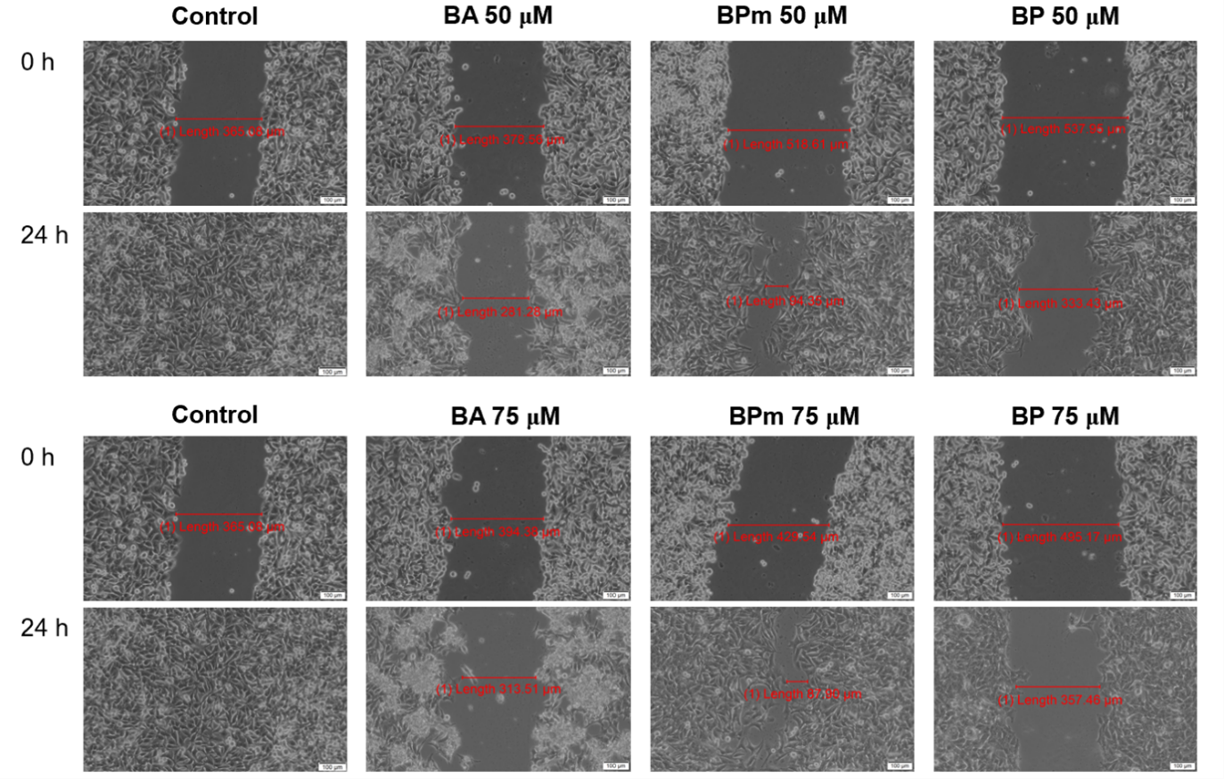

Supplement: Supplementary file 1 [file life-15-01884-s001.zip › Figure S1 Anti-migratory activity of BA, BPm and BP.png]

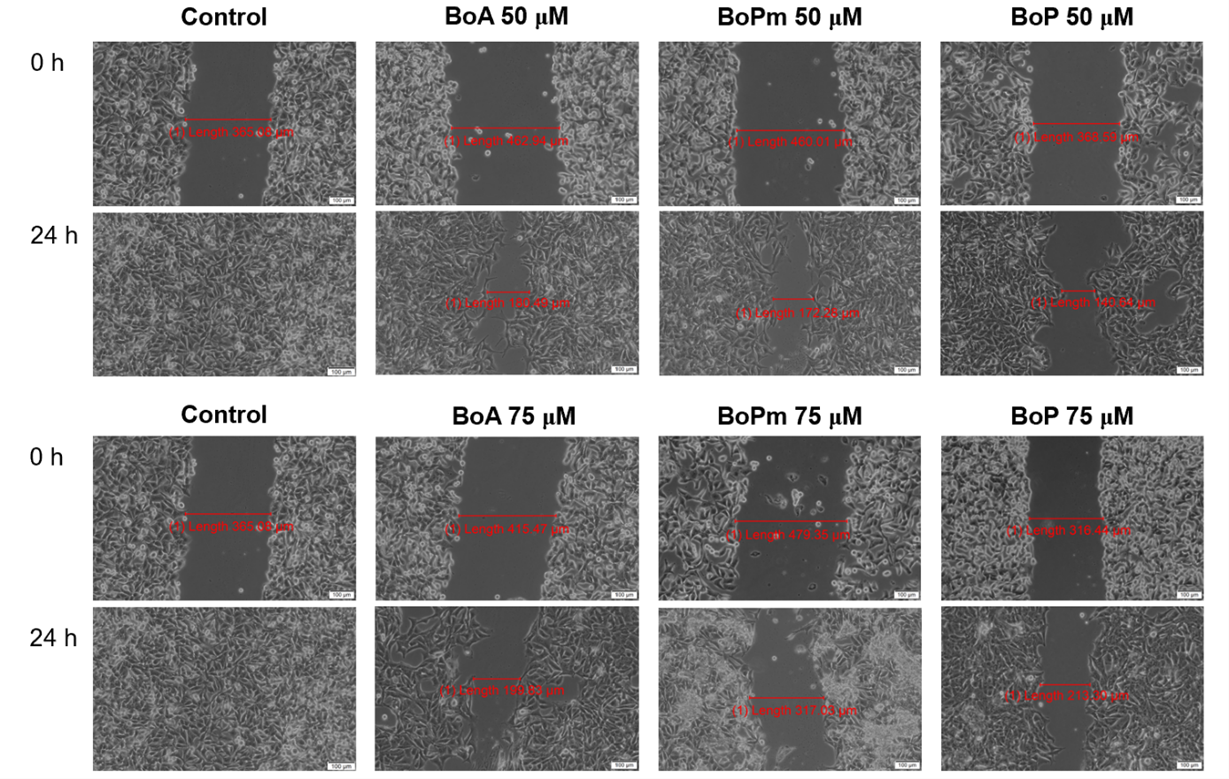

Supplement: Supplementary file 1 [file life-15-01884-s001.zip › Figure S2 Anti-migratory activity of BoA, BoPm and BoP.png]

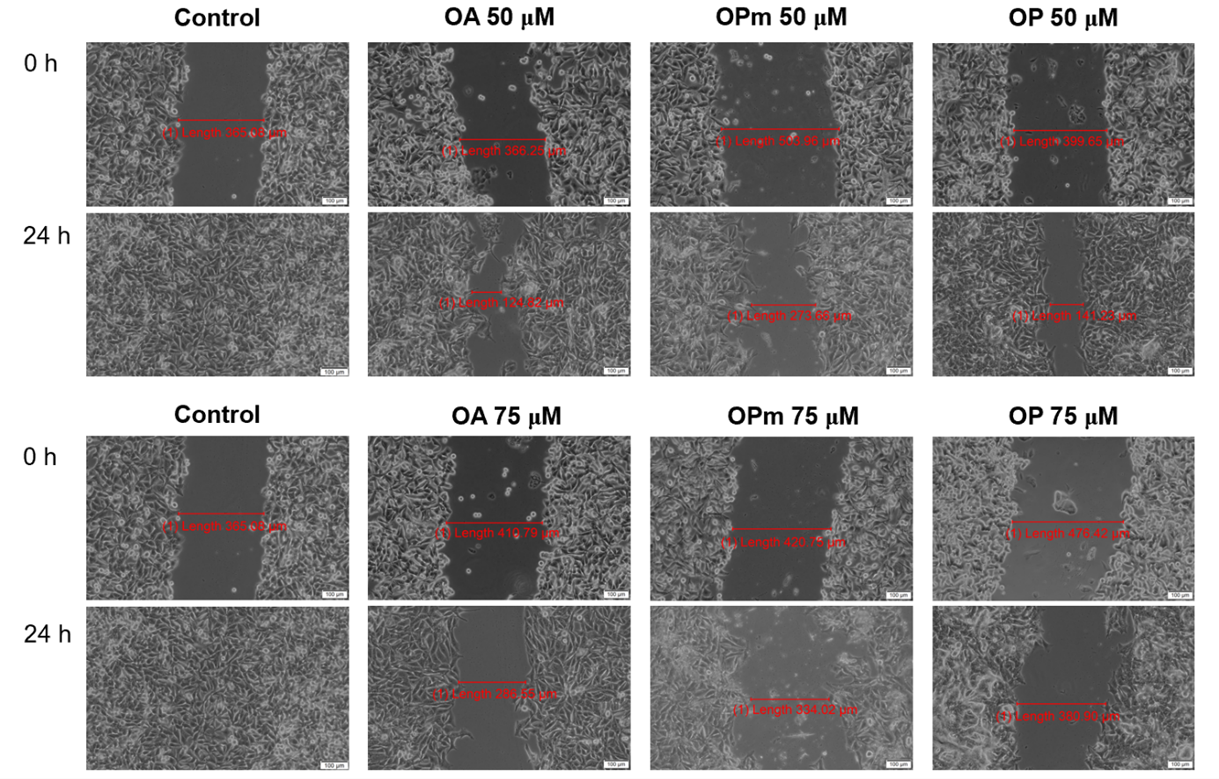

Supplement: Supplementary file 1 [file life-15-01884-s001.zip › Figure S3 Anti-migratory activity of OA, OPm and OP.png]

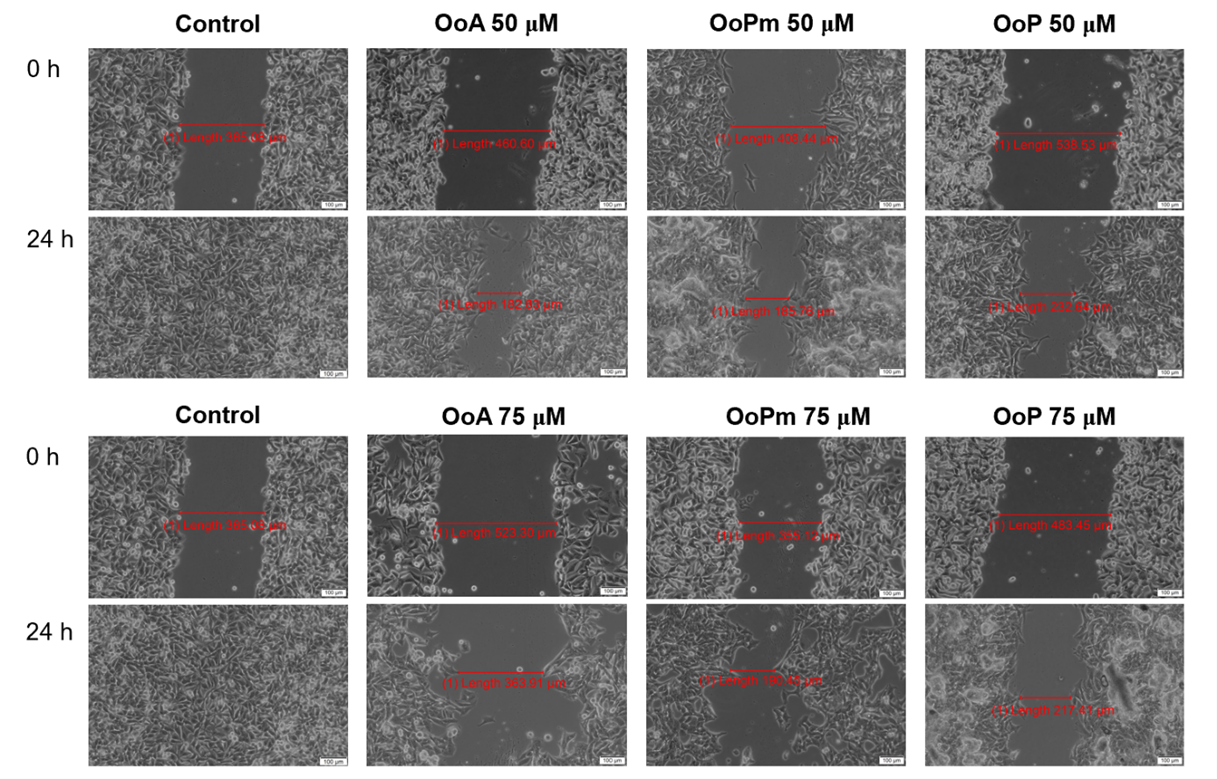

Supplement: Supplementary file 1 [file life-15-01884-s001.zip › Figure S4 Anti-migratory activity of OoA, OoPm and OoP.png]

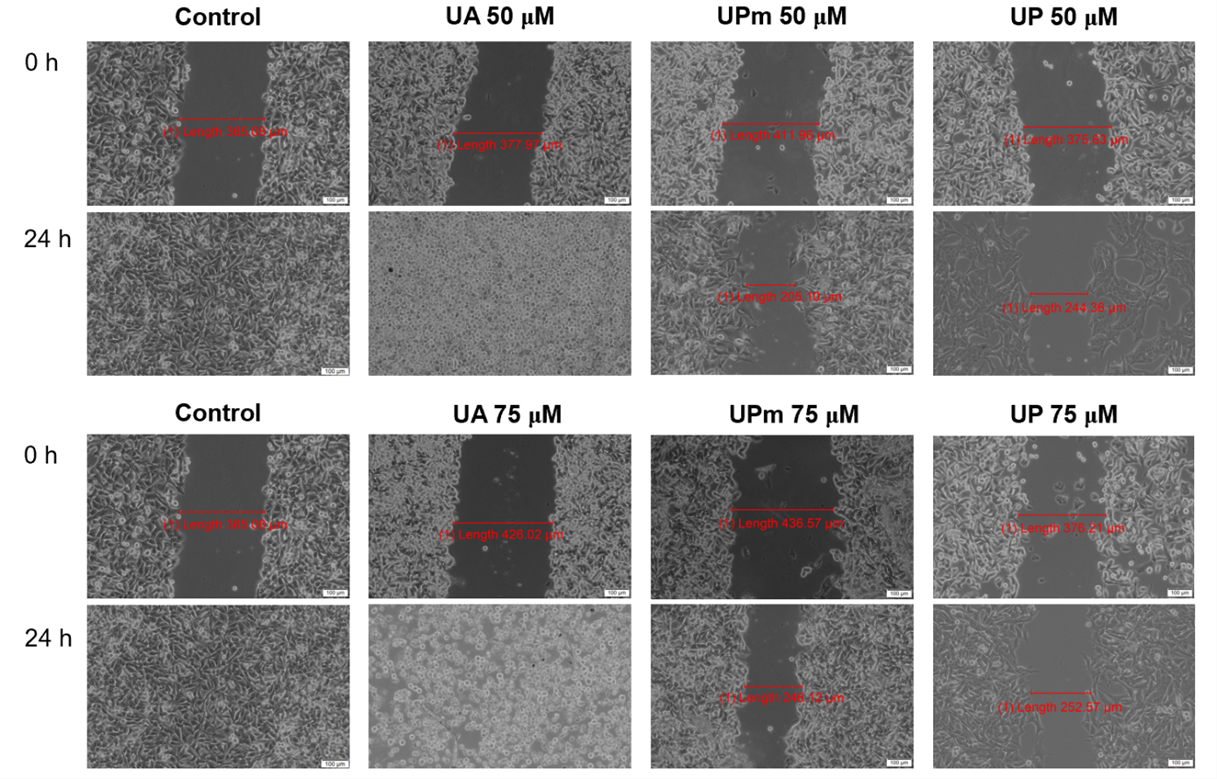

Supplement: Supplementary file 1 [file life-15-01884-s001.zip › Figure S5 Anti-migratory activity of UA, UPm and UP.png]

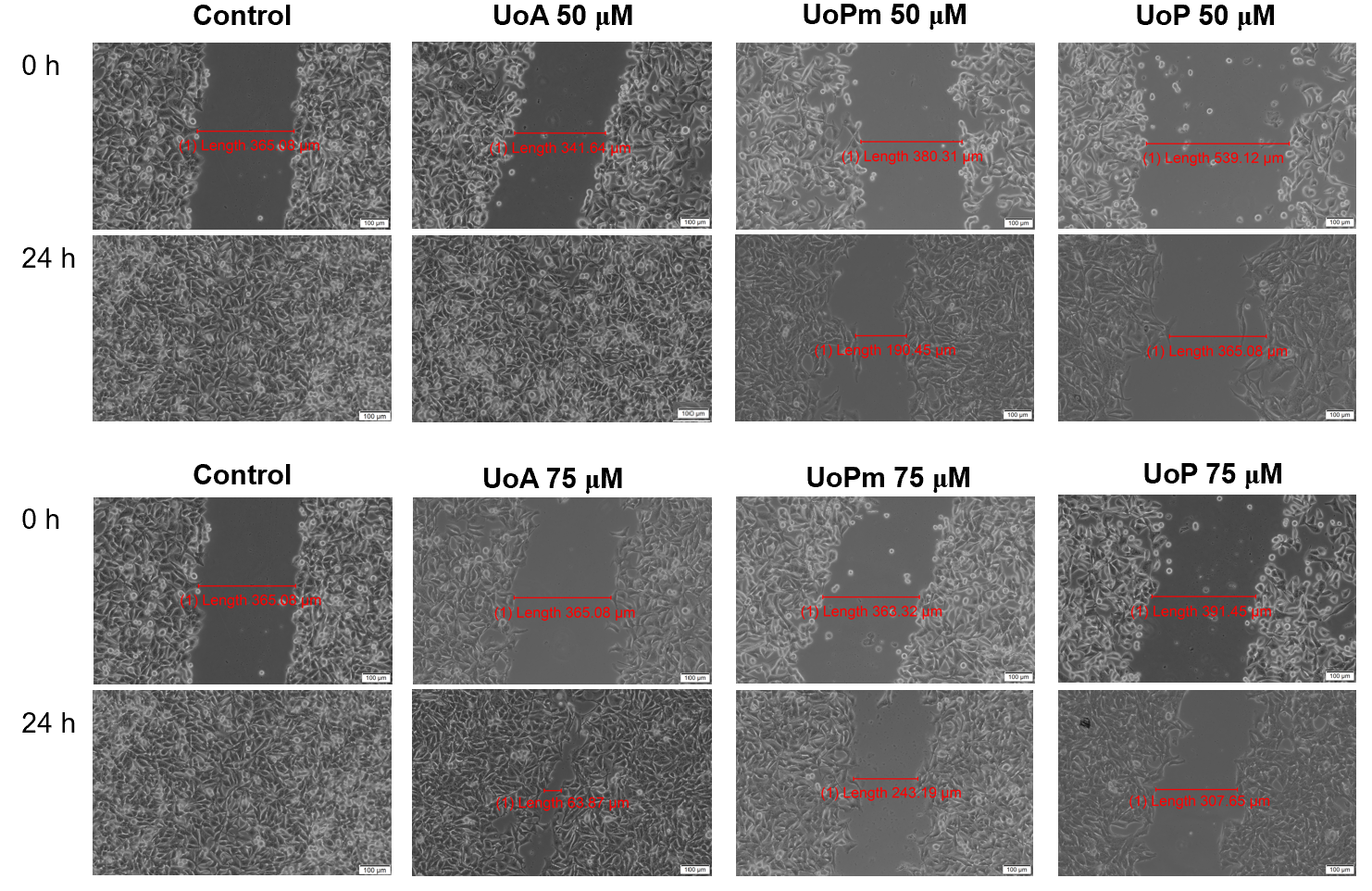

Supplement: Supplementary file 1 [file life-15-01884-s001.zip › Figure S6 Anti-migratory activity of control, UoA, UoPm and Uo.png]
